# Real-World Outcomes of Treatment Approaches and the Impact of Systemic Inflammation Markers on Survival in Patients with Locally Advanced and Metastatic Laryngeal Cancer

**DOI:** 10.3390/jcm14248924

**Published:** 2025-12-17

**Authors:** Burçin Çakan Demirel, Semra Taş, Taliha Güçlü Kantar, Melek Özdemir, Tolga Doğan, Canan Karan, Burcu Yapar Taşköylü, Atike Gökçen Demiray, Serkan Değirmencioğlu, Ahmet Bilici, Gamze Gököz Doğu, Arzu Yaren

**Affiliations:** 1 Department of Medical Oncology, School of Medicine, Medipol University, Istanbul 34214, Türkiye; 2 Department of Medical Oncology, School of Medicine, Pamukkale University, Denizli 20070, Türkiye; 3 Department of Medical Oncology, Denizli State Hospital, Denizli 20070, Türkiyemelekozdemir@hotmail.com.tr (M.Ö.);; 4 Department of Medical Oncology, Tekden Hospital, Denizli 20010, Türkiye; 5 Department of Medical Oncology, Denipol Hospital, Denizli 20010, Türkiye

**Keywords:** laryngeal cancer, Naples prognostic score, systemic inflammation, nutritional status, prognostic biomarkers

## Abstract

**Background:** Systemic inflammation and nutritional status have emerged as promising prognostic indicators across various malignancies; however, their clinical relevance in advanced laryngeal cancer remains underexplored. This study aimed to evaluate the prognostic significance of inflammation- and nutrition-based indices on the overall survival (OS) and progression-free survival (PFS) in patients with locally advanced or metastatic laryngeal cancer. **Methods:** A total of 147 patients treated at Pamukkale University between 2013 and 2022 were retrospectively analyzed. Baseline hematologic and biochemical parameters were used to calculate the Naples Prognostic Score (NPS), the Controlling Nutritional Status (CONUT) score, the Systemic Immune–Inflammation Index (SII), the Systemic Inflammation Response Index (SIRI), the C-reactive Protein/Albumin Ratio (CAR), and the Prognostic Nutritional Index (PNI). Survival outcomes were estimated using the Kaplan–Meier method, and independent prognostic factors were identified by Cox regression analyses. **Results:** The median OS and PFS were 55.5 and 48.8 months, respectively. In univariate analyses, high NPS, CONUT, SIRI, SII, and CAR values were significantly associated with inferior OS and PFS (*p* < 0.05). Multivariate analyses identified advanced stage, disease progression during chemotherapy, and high NPS as independent predictors of both the OS and PFS, whereas surgery conferred a survival advantage. **Conclusions:** Inflammation- and nutrition-based indices, particularly NPS, are strong prognostic markers for survival in patients with advanced laryngeal cancer. Routine integration of these parameters may enhance individualized risk stratification and guide treatment decisions in clinical practice.

## 1. Introduction

Globally, laryngeal cancer accounts for more than 189,000 new cases and over 103,000 deaths annually [1]. By anatomical subsite, glottic tumors comprise approximately two-thirds of cases, supraglottic tumors account for one-third, and subglottic tumors represent 1–2%. Owing to their early symptom presentation, glottic tumors are more frequently detected at earlier stages. Early diagnosis plays a critical role in treatment opportunities and prognosis. In contrast, supraglottic and subglottic tumors generally present with symptoms later on and are often diagnosed at locally advanced or metastatic stages [2,3].

The evaluation of advanced-stage laryngeal cancer patients by multidisciplinary head and neck tumor boards leads to favorable outcomes in terms of prognosis and survival [4,5]. Even systemic treatment decisions benefit from multidisciplinary discussion, primarily because survival rates remain low in locally advanced and advanced disease. Therefore, identifying prognostic factors that are inexpensive, easily accessible, and applicable in daily practice is of great importance. Biomarkers derived from complete blood count (CBC) parameters represent a cost-effective and feasible approach to characterize the tumor microenvironment [6]. A meta-analysis including 49 studies that investigated CBC-derived ratios in head and neck cancer patients demonstrated significant associations with poorer overall survival (OS) [7].

In recent years, multiple studies have shown that composite scores integrating patients’ nutritional status, immune system parameters, and serum protein levels are associated with progression-free survival (PFS) and OS. The Prognostic Nutritional Index (PNI), Controlling Nutritional Status (CONUT), Systemic Immune-Inflammation Index (SII), Systemic Inflammation Response Index (SIRI), Naples Prognostic Score (NPS), and C-reactive Protein/Albumin Ratio (CAR) have all been defined as prognostic parameters reflecting nutritional and immune status [8,9,10,11,12].

Although several inflammation- and nutrition-based biomarkers have been studied in head and neck cancers, evidence specific to laryngeal squamous cell carcinoma (LSCC) remains limited. The current literature indicates that prognostic indices in LSCC are not well established, and available studies are heterogeneous, often combining multiple head and neck subsites, which prevents LSCC-specific conclusions being made [13]. Moreover, recent investigations suggest that systemic inflammatory markers such as NLR and SIRI may have prognostic potential in LSCC, but their predictive performance varies widely across cohorts, indicating inconsistency and limited validation [14]. Importantly, studies evaluating composite inflammation–nutrition scores such as SIRI, SII, NPS, and CONUT simultaneously in LSCC populations are extremely scarce, and no consensus exists regarding their relative prognostic strength [15]. These gaps in the literature highlight the need for focused research investigating accessible, cost-effective biomarkers that may support clinical risk stratification specifically in LSCC.

In this study, we aimed to evaluate the prognostic impact of inflammation- and nutrition-based parameters, which can be derived from routine blood tests, on OS and PFS in patients with locally advanced and metastatic laryngeal cancer.

## 2. Materials and Methods

### 2.1. Patient Data

Between March 2013 and January 2022, pretreatment clinical and laboratory data of patients diagnosed with locally advanced or metastatic laryngeal squamous cell carcinoma (LSCC) who were admitted to the Department of Medical Oncology at Pamukkale University were retrospectively reviewed. A total of 147 consecutive patients met the eligibility criteria and were included in the study. Tumor staging was performed according to the American Joint Committee on Cancer (AJCC) 8th edition. T-stage distribution, including both early-stage (T1–T2) and advanced-stage (T3–T4) disease, was as follows: T1 in 5 patients, T2 in 30 patients, T3 in 58 patients, and T4 in 54 patients. Nodal involvement included N0 in 41 patients, N1 in 71, N2 in 29, and N3 in 6. Metastatic evaluation revealed M0 in 118 patients and M1 in 29 patients. Based on overall AJCC stage grouping, all patients had advanced-stage disease, including 59 patients with Stage III, 56 with Stage IVA, 5 with Stage IVB, and 27 with Stage IVC. No Stage I or Stage II cases were present in the cohort, reflecting a predominantly advanced-stage population typically referred to tertiary oncology centers.

Inclusion Criteria

- Histologically confirmed laryngeal squamous cell carcinoma (LSCC).
- Age ≥ 18 years.
- Availability of complete pretreatment laboratory parameters including full blood count, albumin, CRP, and cholesterol.
- Documented TNM staging (*AJCC 8th* edition).
- Received definitive or systemic oncologic treatment at the participating center.
- Blood samples obtained within 1–7 days before treatment initiation.
- Complete clinical follow-up information available for survival analysis.

Exclusion Criteria

- Active local or systemic infections at the time of blood sampling (acute tonsillitis, sinusitis, dental/periodontal infection, pneumonia, urinary infection).
- Chronic inflammatory or autoimmune diseases (RA, IBD, SLE, sarcoidosis).
- Hematologic malignancy or concurrent active cancer other than LSCC.
- Chronic liver disease, cirrhosis, chronic kidney disease, or severe heart failure.
- Use of corticosteroids, immunosuppressants, or NSAIDs within 2 weeks before blood sampling.
- Laboratory tests not obtained within the standardized pretreatment window.
- Incomplete clinical or biochemical data.

### 2.2. Laboratory Measurements

A total of 147 patients were included. The following laboratory and clinical variables were retrieved from their medical records: age; sex; ECOG performance status (PS); smoking and alcohol history; previous treatments (surgery, radiotherapy, and chemotherapy); disease stage; body mass index (BMI); neutrophil, lymphocyte, monocyte, and platelet counts; mean platelet volume (MPV); serum albumin; C-reactive protein (CRP); and total cholesterol levels. All inflammatory and nutritional biomarkers (NLR, PLR, LMR, SIRI, SII, CONUT, CAR, PNI, and NPS) were calculated using venous blood samples obtained strictly within 1–7 days before initiation of oncologic treatment. CBC parameters were analyzed using the Mindray CAL 8000 (Shanghai, China) autoanalyzer, employing electrical impedance and optical density measurement methods. Serum CRP and albumin levels were measured using the Cobas 702 analyzer (Roche Diagnostics, Mannheim, Germany) based on the electrochemiluminescence method. To ensure analytical consistency, all measurements were performed using standardized automated systems within a single center.

### 2.3. Calculated Parameters

The following prognostic scores were calculated using laboratory values:

- PNI = [Serum albumin (g/dL) × 10] + [Lymphocyte count (/nL) × 0.005].
- SII = (Platelet count × Neutrophil count)/Lymphocyte count.
- SIRI = (Neutrophil count × Monocyte count)/Lymphocyte count.
- NLR = Neutrophil/Lymphocyte.
- PLR = Platelet/Lymphocyte.
- LMR = Lymphocyte/Monocyte.
- CAR = CRP/Albumin.
- BMI = weight (kg)/height^2^ (m^2^).

### 2.4. Nutritional Status Score (CONUT)

The Controlling Nutritional Status (CONUT) score was calculated using three laboratory parameters—serum albumin concentration, total lymphocyte count, and total cholesterol level—reflecting both the nutritional and immunological status of patients. The total CONUT score was obtained by summing these three components, and patients were subsequently classified as follows: normal nutritional status (0–1 points), mildly malnourished (2–4 points), moderately malnourished (5–8 points), or severely malnourished (9–12 points) (Table 1) [16].

### 2.5. Naples Prognostic Score (NPS) Classification

Patients were stratified according to the Naples Prognostic Score (NPS), which integrates serum albumin, total cholesterol, neutrophil-to-lymphocyte ratio (NLR), and lymphocyte-to-monocyte ratio (LMR). Based on the total NPS, participants were divided into three groups for analysis: Group 1 (score = 0), Group 2 (score = 1–2), and Group 3 (score = 3–4) (Table 2) [17].

Ethical approval for the study was obtained from the Non-Interventional Ethics Committee of Pamukkale University (Approval No: E-60116787-020-374745, Date: 1 June 2023).

### 2.6. Statistical Analysis

All statistical analyses were performed using IBM SPSS Statistics for Windows version 25.0 (IBM Corp., Armonk, NY, USA) and GraphPad Prism version 9.0 (GraphPad Software, San Diego, CA, USA). Descriptive statistics were expressed as mean ± standard deviation (SD) or median (range: minimum–maximum) for continuous variables, and as frequency (percentages) for categorical variables. The Kolmogorov–Smirnov test was applied to assess the normality of data distribution. Categorical variables were compared using the Chi-square test or Fisher’s exact test, while continuous variables were analyzed with Student’s *t*-test or the Mann–Whitney U test, depending on the normality of the data.

Receiver operating characteristic (ROC) curve analyses were carried out to determine optimal cut-off points for continuous inflammatory and nutritional indices, including SIRI, SII, CAR, and PNI. The area under the curve (AUC) with 95% confidence intervals (CIs) was calculated to evaluate their discriminatory ability for mortality prediction. Optimal thresholds were identified using the maximum Youden index (sensitivity + specificity −1). The identified cut-off values (SIRI ≥ 1890.62, SII ≥ 909,583.93, CAR ≥ 2.86, and PNI ≤ 48.77) were subsequently used in all survival analyses.

To evaluate the robustness of the ROC-derived thresholds, a sensitivity analysis was performed. For each biomarker, the optimal cut-off value was recalculated with a ±10% variation, and the resulting changes in sensitivity, specificity, and AUC were examined. SIRI, SII, CAR, and PNI demonstrated stable performance across the alternative thresholds, whereas BSA exhibited marked variability. Detailed sensitivity analysis results are provided in Supplementary Table S1.

OS and PFS were estimated using the Kaplan–Meier method, and survival differences between groups were compared by the Log-rank test. Median survival times, together with 2- and 5-year survival rates, were calculated for each subgroup according to clinical and inflammatory–nutritional parameters. Variables showing statistical significance in univariate analyses (*p* < 0.05) were input into a multivariate Cox proportional hazards regression model to identify independent prognostic factors for OS and PFS. The results were expressed as hazard ratios (HRs) and 95% confidence intervals (CIs). The proportional hazards assumption was verified by Schoenfeld residuals. All statistical tests were two-tailed, and a *p*-value < 0.05 was considered statistically significant.

Overall Survival (OS): Time from the diagnosis of locally advanced or metastatic disease to death from any cause.

Progression-Free Survival (PFS): Time from diagnosis to disease progression.

## 3. Results

Our study included 147 patients, comprising 138 men (93.9%) and 9 women (6.1%), with a median age of 61 years (range, 33–86 years). The median follow-up period was 34.6 months (range, 2.9–258.9 months). Patients were classified according to pathological stage, with 40.1% in Stage III and 59.9% in Stage IV disease. The most common primary tumor site was glottic (53.1%), followed by supraglottic (30.6%) and subglottic (16.3%) locations. Surgical treatment was performed in 65.3% of the cohort; among these, 74.4% underwent total laryngectomy, while 25.6% underwent subtotal procedures. Regarding treatment modalities, radiotherapy (RT) (39.5%) was the most frequently applied initial approach, followed by chemotherapy (CT) (33.3%) and concurrent chemoradiotherapy (CRT) (19.0%). Adjuvant therapy was administered to 12.2% of the patients, and induction chemotherapy to 8.8%. In terms of viral biomarkers, HPV positivity was identified in 5.4%, and p16 positivity in 22.4% of evaluable cases. Based on nutritional and inflammatory indices, high-risk features were common: NPS grade 2 (44.2%), CONUT ≥ 3 (34.7%), SIRI ≥ 1890.62 (51.7%), SII ≥ 909,583.93 (52.4%), and CAR ≥ 2.86 (51.7%). During follow-up, disease progression occurred in 62.6% of the patients, and 57.8% had died by the end of the observation period. Detailed demographic and clinical characteristics are summarized in Supplementary Table S2.

The clinical variables presented in Table 3. Specifically, factors associated with more advanced disease burden—such as older age, smoking, alcohol use, advanced stage, and lack of surgery—were similarly linked to shorter OS and PFS. These patterns also paralleled the distribution of inflammatory and nutritional markers: patients with higher NPS, CONUT, SIRI, SII, and CAR values tended to have a more advanced tumor stage and display poorer treatment responses. This alignment between clinical characteristics and biomarker profiles suggests that systemic inflammation and nutritional impairment may partially reflect underlying tumor aggressiveness.

The descriptive statistics of the inflammatory and nutritional indices revealed wide variability. For example, the mean body surface area (BSA) was 1.78 ± 0.18 m^2^ (median 1.77; range, 1.30–2.38); the mean SIRI and SII were 3124.51 ± 3631.02 and 1,540,696.10 ± 1,494,576.91, respectively; the median SIRI was 1965.05 (range 150.38–29,458.33) and median SII 994,170.98 (range 96,075.95–76,977,330.77); and the mean CAR was 9.74 ± 25.67 (median 3.06; range 0.07–290.00), and mean PNI 48.93 ± 7.10 (median 48.15; range 31.10–64.15). These findings reflect the heterogeneous clinical and metabolic profiles of patients with laryngeal cancer.

The median overall survival (OS) was 55.53 months (95% CI: 27.90–82.15); 2- and 5-year OS rates were 71.5% and 48.7%, respectively. OS differed significantly according to age (*p* = 0.005), smoking status (*p* = 0.042), alcohol use (*p* = 0.046), surgery (*p* = 0.007), stage (*p* = 0.002), adjuvant therapy (*p* = 0.048), and chemotherapy response (*p* < 0.001). Patients aged ≤ 65 years, those undergoing surgery, and those receiving adjuvant therapy lived longer, whereas Stage IV disease and poor chemotherapy response were associated with shorter OS. Among the inflammation–nutrition indices, higher NPS (*p* < 0.001), CONUT (*p* = 0.001), SIRI (*p* < 0.001), SII (*p* < 0.001), and CAR (*p* = 0.001) were linked to reduced OS, while a higher PNI showed a non-significant trend with better outcomes. The OS-related findings are summarized in Table 4.

Across the cohort, elevated systemic inflammatory and nutritional markers were more frequently observed in patients with advanced-stage disease, those who did not undergo surgery, and those who demonstrated progressive disease after chemotherapy. Patients requiring second-line treatment also exhibited markedly higher SIRI, SII, NPS, CONUT, and CAR levels. These patterns suggest that systemic inflammation is influenced not only by tumor biology but also by disease burden and treatment responsiveness, providing important context for interpreting the prognostic performance of these indices.

**Figure 1:**
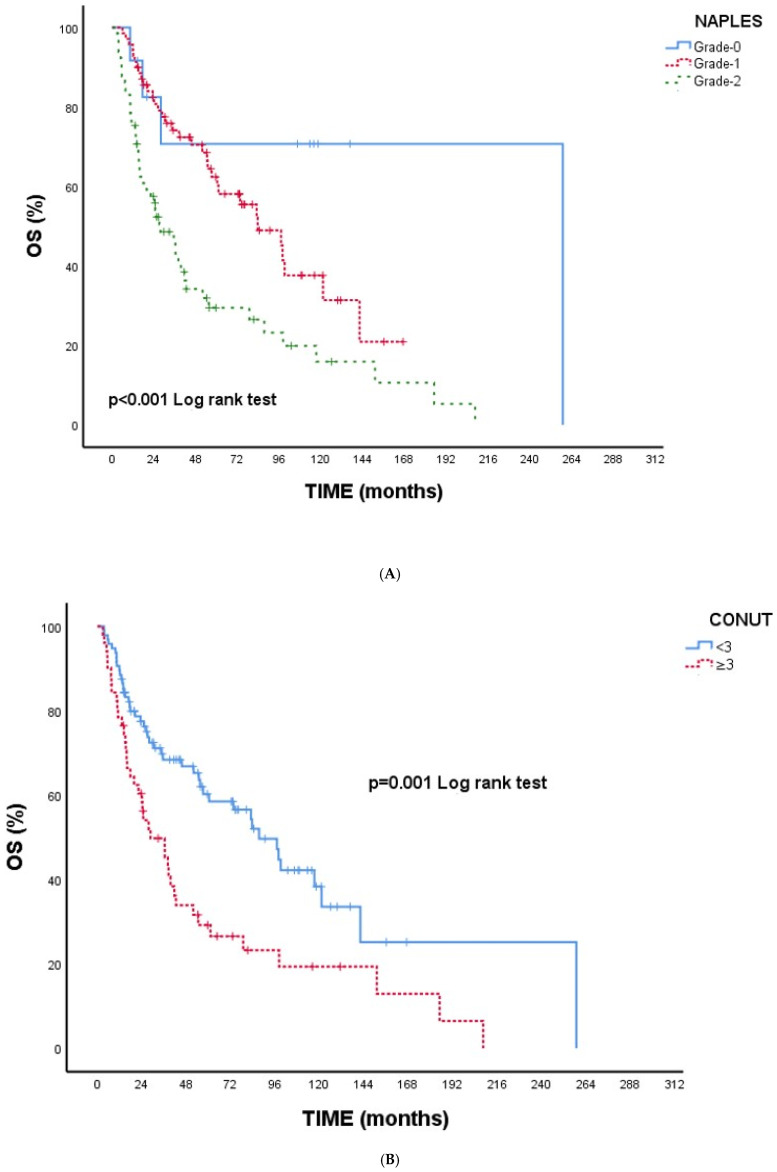

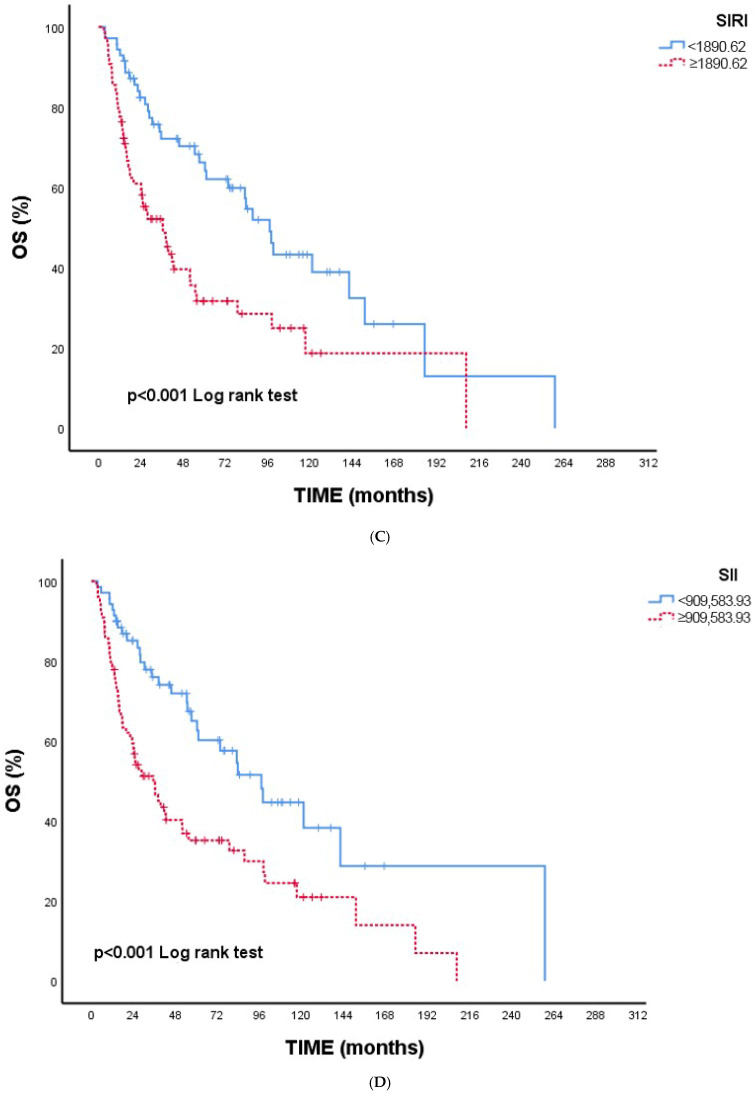

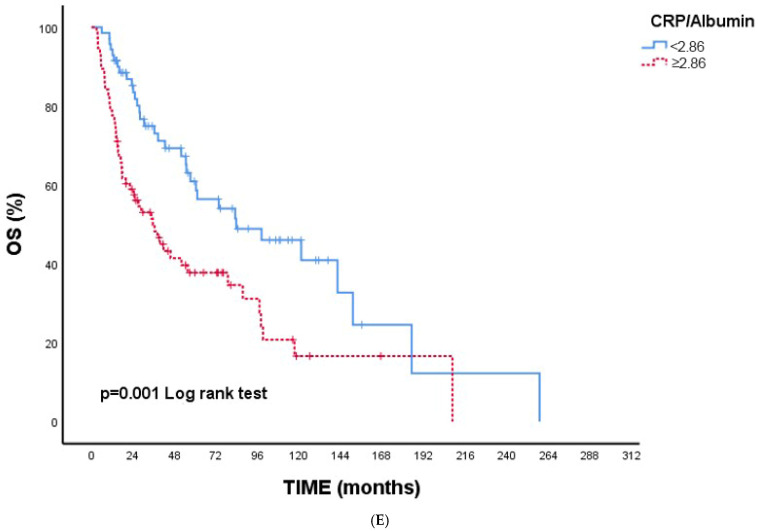
(**A**) Overall survival according to NPS. (**B**) Overall survival according to CONUT. (**C**) Overall survival according to SIRI. (**D**) Overall survival according to SII. (**E**) Overall survival according to CAR. **Abbreviations:** NPS—Naples Prognostic Score; CONUT—Controlling Nutritional Status; SIRI—Systemic Inflammation Response Index; SII—Systemic Immune–Inflammation Index; CAR—C-reactive protein/albumin ratio.

**Figure 1A–E Kaplan–Meier overall survival curves according to inflammatory and nutritional indices**. Figure 1A **NPS**: Higher NPS grades were associated with significantly reduced OS (*p* < 0.001). Figure 1B **CONUT**: Patients with CONUT ≥ 3 demonstrated worse OS (*p* = 0.001). Figure 1C **SIRI**: Elevated SIRI (≥1890.62) was associated with poorer OS (*p* < 0.001). Figure 1D **SII**: Higher SII (≥909,583.93) predicted significantly shorter OS (*p* < 0.001). Figure 1E **CAR**: CAR ≥2.86 was linked to worse OS (*p* = 0.001).

**Overall, all indices were consistently indicative of poorer survival with higher inflammatory or nutritional impairment, supporting the univariate findings (Table 4)**.

The median PFS was 48.83 months (95% CI: 30.15–67.50), with 2- and 5-year survival rates of 63.4% and 43.3%, respectively. Significant differences in PFS were observed according to age (*p* = 0.026), smoking status (*p* = 0.001), alcohol consumption (*p* = 0.032), surgery (*p* = 0.008), disease stage (*p* = 0.003), chemotherapy response (*p* < 0.001), second-line chemotherapy (*p* < 0.001), and inflammation–nutrition indices including NPS (*p* = 0.001), CONUT (*p* = 0.003), SIRI (*p* = 0.001), SII (*p* = 0.003), and CAR (*p* < 0.001). Patients aged ≤65 years, smokers, and those undergoing surgery demonstrated significantly longer PFS. Similarly, patients with Stage III disease, who displayed stable or complete chemotherapy response, and had no need for second-line chemotherapy had superior outcomes. Conversely, patients with higher NPS, CONUT, SIRI, SII, and CAR values showed shorter PFS durations, underscoring the strong prognostic value of systemic inflammation and nutritional status in predicting disease progression among laryngeal cancer patients. The findings related to PFS are summarized in Table 5.

The results of the multivariate Cox regression for mortality risk are summarized in Table 6. Independent predictors of increased mortality were Stage IV disease (HR = 3.71; 95% CI: 1.76–7.84; *p* = 0.001), disease progression in response to chemotherapy (HR = 3.21; 95% CI: 1.19–8.65; *p* = 0.021), and higher NPS grades—Grade 1 (HR = 7.66; 95% CI: 1.56–37.63; *p* = 0.012) and Grade 2 (HR = 12.74; 95% CI: 1.94–83.52; *p* = 0.008). Also, surgery reduced mortality risk (HR = 0.48; 95% CI: 0.25–0.91; *p* = 0.025).

The results of the multivariate Cox regression analysis for progression risk are presented in Table 7. Independent predictors of shorter PFS included Stage IV disease (HR = 2.19; 95% CI: 1.11–4.34; *p* = 0.024), disease progression in response to chemotherapy (HR = 3.25; 95% CI: 1.31–8.08; *p* = 0.011), and higher NPS grades, specifically Grade 1 (HR = 3.79; 95% CI: 1.16–12.40; *p* = 0.027) and Grade 2 (HR = 8.05; 95% CI: 1.75–37.05; *p* = 0.007). In contrast, surgery (HR 0.33; 95% CI 0.17–0.64; *p* = 0.001) and the absence of second-line chemotherapy (HR = 0.37; 95% CI: 0.18–0.78; *p* = 0.009) were independently associated with a reduced risk of progression. These findings highlight that disease stage, treatment response, surgical management, and the NPS index are significant prognostic factors influencing PFS in patients with laryngeal cancer.

## 4. Discussion

The present study provides a comprehensive evaluation of multiple inflammation- and nutrition-based prognostic indices in patients with laryngeal cancer, demonstrating clear and consistent associations between these biomarkers and survival outcomes. Among all of the evaluated indices, NPS showed the strongest prognostic performance, maintaining independent significance for both OS and PFS after adjustment for clinical variables. CONUT, SIRI, SII, and CAR also exhibited meaningful stratification in univariate analyses, further supporting their relevance as markers of host inflammatory and nutritional status. These patterns closely parallel the Kaplan–Meier estimations and the multivariate Cox models presented in Section 3, reinforcing the concept that elevated systemic inflammation and impaired nutrition contribute to poorer prognosis. By integrating our findings with the existing literature, this discussion contextualizes the prognostic capacity of these indices and underscores their potential clinical implications.

Consistent with previous literature, our study demonstrated that higher CONUT scores were significantly associated with poorer OS and PFS, reflecting the adverse prognostic impact of impaired nutritional and immunological status in laryngeal cancer. Although CONUT showed strong discrimination in the univariate analyses, it did not remain an independent predictor in the multivariate model, suggesting that its prognostic value may be partially mediated through factors such as tumor stage, systemic inflammatory burden, and comorbidity-related nutritional decline. This pattern has also been observed in prior meta-analyses evaluating CONUT and GNRI across multiple malignancies, in which these indices correlated strongly with survival outcomes but occasionally lost statistical independence when adjusted for major clinicopathological variables [18].

Recent studies specifically examining laryngeal carcinoma further support our findings: elevated CONUT scores have been associated with advanced stage, increased tumor burden, and inferior OS and DFS following curative surgery [19]. Similarly, both CONUT and PNI have been identified as reliable prognostic indicators in head and neck cancers, including laryngeal tumors [20]. Taken together, these results highlight that nutritional depletion—captured by CONUT—plays a meaningful role in shaping prognosis, even when overshadowed by more dominant factors in multivariable models.

In contrast, the Prognostic Nutritional Index (PNI) did not reach statistical significance in our cohort, possibly due to the predominance of advanced-stage disease, in which systemic inflammatory burden may outweigh isolated nutritional parameters. Additionally, because PNI is mainly based on serum albumin and lymphocyte counts, it may be influenced by acute-phase responses and treatment-related factors, limiting its prognostic discrimination in real-world populations. This reinforces the multidimensional nature of prognostic assessment in laryngeal cancer and underscores the complementary value of composite indices such as NPS, which integrate both nutritional and inflammatory domains into a single prognostic framework.

Additional evidence from recent head and neck cancer research further strengthens the prognostic relevance of the CONUT system. A large meta-analysis including patients with various head and neck subsites demonstrated that higher CONUT scores were consistently associated with poorer OS and DFS, as well as with adverse pathological characteristics such as advanced T/N stage and greater tumor aggressiveness [21]. Moreover, it has recently been demonstrated in a large cohort of surgically treated head and neck squamous cell carcinoma that CONUT effectively stratified survival outcomes and significantly improved prognostic accuracy when incorporated into clinicopathological nomograms, outperforming AJCC staging alone [22]. Furthermore, a dedicated cohort study focusing exclusively on laryngeal squamous cell carcinoma demonstrated that CONUT, together with PNI and GPS, provided strong prognostic stratification for overall and disease-free survival, and significantly enhanced prognostic accuracy when evaluated alongside traditional clinicopathological variables [23]. Collectively, these findings highlight that CONUT represents a robust and reproducible biomarker across head and neck malignancies, aligning with the patterns observed in our cohort.

The NPS, which integrates both nutritional and inflammatory components, provides a multidimensional reflection of the host’s physiological status. Its prognostic value has been validated in population-based analyses [11], and several studies in oral cavity and hypopharyngeal squamous cell carcinoma have reported NPS as an independent predictor of overall and disease-free survival, with higher scores consistently indicating poorer outcomes [24,25]. Furthermore, prognostic models incorporating NPS alongside clinicopathological variables have demonstrated superior predictive accuracy compared with the AJCC staging system alone, effectively stratifying patients into distinct risk groups [26]. In accordance with these observations, our findings showed that higher NPS was independently associated with both reduced OS and PFS in laryngeal cancer. The strong and persistent prognostic effect of NPS in our multivariate analysis suggests that it may better capture the interplay between systemic inflammation, nutritional decline, and tumor biology, underscoring its potential utility as a comprehensive biomarker for individualized risk assessment and treatment planning in head and neck malignancies.

Systemic inflammation-based biomarkers have emerged as important prognostic indicators in head and neck cancers. In our study, elevated SII and SIRI values were strongly associated with shorter OS and PFS, underscoring their ability to reflect tumor aggressiveness. These findings are consistent with recent evidence showing that higher SII levels predict poorer survival outcomes in head and neck cancer patients [27], and this association is further supported by meta-analytic data confirming the adverse prognostic impact of SII [28]. Similarly, the prognostic relevance of SIRI has been demonstrated in oral cavity squamous cell carcinoma, where propensity score-based analyses have identified elevated SIRI as an independent predictor of mortality, and this has been validated in large head and neck squamous cell carcinoma cohorts, showing its capacity to capture systemic inflammatory burden associated with worse outcomes [29,30]. In our cohort, higher SII and SIRI values paralleled adverse clinical features—including advanced disease stage, lack of surgery, progressive chemotherapy response, and the need for second-line treatment—suggesting that systemic inflammation reflects not only tumor biology but also disease burden and treatment responsiveness. Collectively, these findings support the integration of inflammation-based indices such as SII and SIRI into routine prognostic evaluation for patients with laryngeal cancer.

Consistent with our findings, previous studies have shown that inflammation-based markers reflecting both systemic inflammatory activity and nutritional status hold prognostic relevance in head and neck cancers. Among these, the CAR has emerged as a robust indicator of adverse outcomes. Studies in laryngeal and hypopharyngeal squamous cell carcinoma have demonstrated that elevated CAR is significantly associated with advanced tumor burden, higher recurrence rates, and poorer survival [31,32]. A recent meta-analysis further confirmed that elevated CRP levels independently predict worse overall survival across head and neck squamous cell carcinomas, underscoring the strong link between systemic inflammation and tumor aggressiveness [33]. In line with this evidence, our cohort showed that a higher CAR was significantly associated with reduced OS and PFS, supporting its potential role as an accessible and clinically meaningful prognostic biomarker in laryngeal cancer.

The biological heterogeneity of head and neck cancers should also be considered when interpreting inflammation-based biomarkers. HPV-driven carcinogenesis is predominantly associated with oropharyngeal tumors, whereas it is considerably less common in laryngeal cancer. Previous studies have shown that systemic inflammatory ratios such as NLR, PLR, and SII may exhibit stronger prognostic relevance in HPV-positive head and neck cancers [34]. However, HPV/p16 testing was not routinely performed in our cohort; therefore, the potential modifying effect of HPV status on the inflammatory biomarkers’ prognostic performance could not be assessed. This limitation should be taken into account when interpreting our findings.

This study possesses several notable strengths. Specifically, it provides one of the most comprehensive evaluations of multiple inflammation- and nutrition-based biomarkers in laryngeal cancer, integrating NPS, CONUT, SII, SIRI, and CAR within a single, uniformly analyzed cohort. The combined use of Kaplan–Meier survival estimations and multivariate Cox regression strengthened the robustness of the prognostic assessments. Furthermore, the real-world nature of the dataset, inclusion of clinically meaningful outcomes—such as chemotherapy response and second-line treatment requirements—and the long follow-up duration enhance the applicability of the findings. By examining the interplay between systemic inflammation, nutritional status, and tumor aggressiveness, this study offers clinically relevant insights that extend beyond traditional prognostic factors.

However, several limitations should be acknowledged. The retrospective, single-center design may have introduced selection bias and restricted generalizability. The cut-off values for the inflammatory and nutritional indices were derived from this cohort and may not be universally applicable, emphasizing the need for external validation. The biomarkers were assessed only at baseline, preventing an analysis of dynamic changes in systemic inflammation or nutritional status over time. Additionally, HPV/p16 status was not routinely available, limiting the ability to evaluate its potential modifying effect on biomarker performance. Unmeasured confounders such as comorbidities and treatment heterogeneity may have also influenced the results. Finally, molecular or genomic markers were not incorporated, which may have further refined risk stratification. Prospective multicenter studies are needed to validate these findings and establish the clinical utility of these indices in laryngeal cancer.

## 5. Conclusions

This study demonstrates that inflammation- and nutrition-based biomarkers provide meaningful prognostic information in laryngeal cancer, complementing traditional clinicopathological factors. Among all of the indices evaluated, NPS showed the strongest independent prognostic performance, while CONUT, SII, SIRI, and CAR also exhibited clear survival stratification and mirrored adverse clinical features such as an advanced stage, poor treatment responses, and the need for second-line therapy. These findings suggest that systemic inflammation and nutritional impairment reflect underlying tumor aggressiveness and may assist in identifying high-risk patients who require closer monitoring or more intensive treatment strategies. Incorporating these biomarkers into routine assessments could enhance risk stratification in laryngeal cancer. Prospective multicenter studies with longitudinal biomarker evaluation are warranted to validate these results and define their potential role in guiding individualized treatment planning.

## Figures and Tables

**Table 1:** Calculation of the CONUT Score.

Parameters	Normal (0)	Mild (1–2)	Moderate (4–6)	Severe (≥7)
Serum albumin (g/dL)	≥3.5 (0)	3.0–3.49 (2)	2.5–2.99 (4)	<2.5 (6)
Total lymphocyte count (/mm^3^)	≥1600 (0)	1200–1599 (1)	800–1199 (2)	<800 (3)
Total cholesterol (mg/dL)	≥180 (0)	140–179 (1)	100–139 (2)	<100 (3)
Total score	0–1	2–4	5–8	9–12

**Table 2:** Calculation of the NPS.

Parameters	Values	Score
Serum albumin (g/dL)	≥4; <4	0/1
Total cholesterol (mg/dL)	>180; ≤180	0/1
NLR	≤2.96; >2.96	0/1
LMR	>4.44; ≤4.44	0/1

**Table 3:** Distribution of Sociodemographic and Clinical Variables.

Variables	N	%
**Age**		
Mean ± SD	61.00 ± 9.83	
Median (min–max)	61.0 (33–86)	
≤65	98	66.7
>65	49	33.3
**Gender**		
Female	9	6.1
Male	138	93.9
**Tumor Location**		
Supraglottic	45	30.6
Glottic	78	53.1
Subglottic	24	16.3
**Smoking**		
No	7	4.8
Yes	140	95.2
**Alcohol**		
No	123	83.7
Yes	24	16.3
**Surgery**		
No	51	34.7
Yes	96	65.3
**HPV**		
Negative	22	15.0
Positive	8	5.4
Unknown	117	79.6
**P16**		
Negative	14	9.5
Positive	33	22.4
Unknown	100	68.0
**Stage**		
III	59	40.1
IV	88	59.9
**NPS**		
Grade-0	12	8.2
Grade-1	70	47.6
Grade-2	65	44.2
**CONUT**		
<3	96	65.3
≥3	51	34.7
**SIRI**		
<1890.62	71	48.3
≥1890.62	76	51.7
**SII**		
<909,583.93	70	47.6
≥909,583.93	77	52.4
**CAR**		
<2.86	71	48.3
≥2.86	76	51.7
**PNI**		
>48.77	71	48.3
≤48.77	76	51.7
**Progression**		
No	55	37.4
Yes	82	62.6
**Mortality**		
Alive	62	42.2
Deceased	85	57.8
**Follow-up time (months)**		
Mean ± SD	50.79 ± 46.24	
Median (min–max)	34.57 (2.87–258.87)	

**Table 4:** Univariate analysis of clinicopathological and systemic inflammation- and nutrition-based parameters for OS.

Variables	2-Year OS (%)	5-Year OS (%)	Median OS (95% CI)	*p*-Value
Overall	71.5	48.7	55.53 (27.90–82.15)	
**Age**				**0.005**
≤65	71.7	57.4	97.03 (59.61–134.44)	
>65	64.1	30.2	38.00 (25.57–50.42)	
**Gender**				0.394
Female	44.4	33.3	20.30 (12.35–28.30)	
Male	73.3	49.6	57.10 (33.33–80.86)	
**Tumor location**				0.993
Supraglottic	70.4	49.4	45.57 (15.03–76.10)	
Glottic	73.7	47.6	54.80 (25.11–84.48)	
Subglottic	66.4	50.2	83.53 (10.62–156.43)	
**Smoking**				**0.042**
No	42.9	14.3	20.33 (13.32–27.33)	
Yes	73.0	50.1	60.30 (36.07–84.52)	
**Alcohol**				**0.046**
No	69.3	45.0	51.97 (33.52–70.41)	
Yes	78.4	67.6	121.17 (49.44–192.89)	
**Surgery**				**0.007**
No	51.4	36.0	24.03 (11.98–36.07)	
Yes	82.0	55.4	82.93 (51.74–114.11)	
**Surgery Type**				0.772
Total	84.3	53.1	61.03 (16.46–105.41)	
Subtotal	74.5	59.2	83.53 (25.71–141.34)	
**HPV**				0.652
Negative	59.1	47.4	54.43 (8.17–100.68)	
Positive	62.5	62.5	60.30 (0.00–151.77)	
Unknown	74.4	47.9	55.53 (20.81–90.24)	
**P16**				0.597
Negative	71.4	54.4	61.03 (23.55–98.50)	
Positive	72.6	56.6	60.30 (5.85–114.74)	
Unknown	71.2	45.4	51.97 (19.35–84.58)	
**Stage**				**0.002**
III	84.6	65.3	99.13 (62.14–136.12)	
IV	62.5	35.9	34.57 (22.98–46.15)	
**Initial treatment**				0.440
None	66.7	55.6	73.50 (0.00–170.70)	
CT	64.3	41.7	45.57 (18.09–73.05)	
CRT	61.9	50.4	61.03 (6.26–115.79)	
RT	82.7	53.8	78.77 (44.90–112.63)	
**Induction therapy**				0.845
Received	65.8	65.8	97.03 (-)	
Not received	71.9	48.0	55.53 (29.30–81.75)	
**Adjuvant therapy**				**0.048**
Received	88.9	80.8	– (–)	
Not received	69.1	44.7	51.97 (35.83–68.10)	
**Chemotherapy response**				**<0.001**
Stable disease	81.6	66.9	185.00 (-)	
Progressive disease	47.3	19.1	17.60 (7.13–28.06)	
Mixed response	-	-	82.93 (-)	
Complete response	96.3	66.3	(–)	
Partial response	43.8	29.2	19.83 (14.52–25.13)	
**Second-line CT**				0.154
Received	81.8	41.5	39.50 (21.21–51.78)	
Not received	68.6	51.4	73.50 (36.37–110.62)	
**NPS**				**<0.001**
Grade-0	82.5	70.7	258.87 (-)	
Grade-1	82.4	62.4	83.53 (58.27–108.78)	
Grade-2	57.5	29.5	27.63 (14.16–41.09)	
**CONUT**				**0.001**
<3	77.5	60.3	83.37 (61.77–112.97)	
≥3	60.4	29.2	28.73 (14.20–43.25)	
**SIRI**				**<0.001**
<1890.62	82.4	68.3	97.03 (80.51–113.54)	
≥1890.62	61.0	31.7	36.43 (22.06–50.79)	
**SII**				**<0.001**
<909,583.93	85.2	65.0	97.03 (68.31–125.74)	
≥909,583.93	59.4	35.1	35.33 (21.31–49.34)	
**CAR**				**0.001**
<2.86	85.2	60.9	83.53 (29.13–137.92)	
≥2.86	59.0	37.8	35.33 (20.00–50.65)	
**PNI**				0.144
>48.77	77.9	54.6	73.50 (45.81–101.18)	
≤48.77	65.4	41.7	51.77 (33.90–69.63)	

Kaplan–Meier, Log-rank test, *p* < 0.05 was considered statistically significant.

**Table 5:** Univariate analysis of clinicopathological and systemic inflammation- and nutrition-based parameters for PFS.

Variables	2-Year %	5-Year %	Median (95% CI)	*p*
Overall	63.4	43.3	48.83 (30.15–67.50)	
**Age**				**0.026**
≤65	66.6	50.4	66.73 (26.92–106.53)	
>65	56.5	27.2	29.23 (13.67–44.78)	
**Gender**				0.051
Female	44.4	22.2	15.57 (1.25–29.88)	
Male	64.6	44.8	53.90 (35.76–72.03)	
**Tumor location**				0.783
Supraglottic	61.2	42.7	38.00 (7.27–68.72)	
Glottic	69.1	44.4	54.43 (32.52–76.30)	
Subglottic	50.0	39.8	23.63 (0.00–66.69)	
**Smoking**				**0.001**
No	28.6	-	15.57 (2.99–28.14)	
Yes	65.3	45.8	54.43 (30.03–78.82)	
**Alcohol**				**0.032**
No	61.4	39.0	38.00 (16.60–59.39)	
Yes	74.2	64.9	121.17 (49.21–193.12)	
**Surgery**				**0.008**
No	50.9	32.0	24.03 (9.83–38.22)	
Yes	70.0	49.1	57.10 (25.87–88.32)	
**Surgery Type**				0.856
Total	71.1	46.2	54.80 (12.68–96.91)	
Subtotal	65.8	55.7	78.77 (0.00–162.48)	
**HPV**				0.896
Negative	54.5	42.1	53.90 (5.25–102.54)	
Positive	62.5	41.7	34.67 (0.00–76.75)	
Unknown	65.2	43.3	41.60 (19.64–63.55)	
**P16**				0.668
Negative	63.5	44.4	53.90 (0.00–128.03)	
Positive	66.5	46.9	55.53 (6.63–104.42)	
Unknown	62.4	41.8	38.00 (13.98–62.02)	
**Stage**				**0.003**
III	77.8	60.5	87.37 (54.79–119.95)	
IV	53.0	29.6	26.87 (20.11–33.62)	
**Initial treatment**				0.465
None	66.7	55.6	73.50 (0.00–170.70)	
CT	53.9	37.2	26.87 (5.72–48.01)	
CRT	65.3	49.5	53.90 (0.00–112.14)	
RT	70.5	44.7	51.77 (29.13–74.40)	
**Induction therapy**				0.646
Received	51.9	51.9	97.03 (-)	
Not received	62.8	43.1	48.83 (30.56–67.09)	
**Adjuvant therapy**				0.110
Received	76.9	62.2	97.03 (-)	
Not received	61.6	40.8	38.00 (15.87–60.12)	
**Chemotherapy response**				**<0.001**
Stable disease	75.3	54.9	184.00 (-)	
Progressive disease	21.4	9.5	11.07 (7.25–14.88)	
Mixed response	-	-	80.23 (-)	
Complete response	91.9	61.9	(-)	
Partial response	46.9	31.3	15.30 (0.00–39.39)	
**Second-line CT**				**<0.001**
Received	45.5	18.2	23.63 (17.77–29.48)	
Not received	69.3	52.0	78.70 (41.01–116.52)	
**NPS**				**0.001**
Grade-0	72.9	60.8	118.30 (0.00–282.64)	
Grade-1	75.2	54.1	73.50 (45.51–101.48)	
Grade-2	48.4	27.8	23.77 (15.22–32.31)	
**CONUT**				**0.003**
<3	70.6	53.9	80.23 (49.07–111.38)	
≥3	50.4	25.3	24.03 (13.22–34.83)	
**SIRI**				**0.001**
<1890.62	74.7	58.4	81.87 (50.03–113.70)	
≥1890.62	52.7	28.9	25.03 (13.58–36.47)	
**SII**				**0.003**
<909,583.93	79.0	55.1	80.23 (47.55–112.90)	
≥909,583.93	49.9	33.2	23.77 (17.49–30.04)	
**CAR**				**<0.001**
<2.86	80.6	54.0	80.23 (21.23–139.22)	
≥2.86	47.8	33.5	23.63 (14.65–32.60)	
**PNI**				0.260
>48.77	69.0	46.2	54.80 (13.78–95.81)	
≤48.77	58.3	40.6	41.60 (12.15–71.04)	

Kaplan–Meier curve, Log-rank test, *p* < 0.05 was considered statistically significant.

**Table 6:** Multivariate Cox regression results of mortality risk according to various clinical variables.

Variables	HR (95% CI)	*p*
**Age**		0.525
≤65	ref	
>65	1.23 (0.64–2.36)	
**Smoking**		0.318
No	ref	
Yes	0.60 (0.22–1.62)	
**Alcohol**		0.803
No	ref	
Yes	0.88 (0.34–2.26)	
**Surgery**		**0.025**
No	ref	
Yes	0.48 (0.25–0.91)	
**Stage**		**0.001**
III	ref	
IV	3.71 (1.76–7.84)	
**Adjuvant therapy**		0.627
Received	ref	
Not received	1.30 (0.44–3.78)	
**Chemotherapy response**		**0.003**
Stable disease	ref	
Progressive disease	3.21 (1.19–8.65)	**0.021**
Mixed response	1.873 (0.31–11.45)	0.494
Complete response	0.58 (0.19–1.79)	0.346
Partial response	2.36 (0.64–8.66)	0.195
**NPS**		**0.027**
Grade-0	ref	
Grade-1	7.66 (1.56–37.63)	**0.012**
Grade-2	12.74 (1.94–83.52)	**0.008**
**CONUT**		0.752
<3	ref	
≥3	0.87 (0.37–2.03)	
**SIRI**		0.937
<1890.62	ref	
≥1890.62	1.03 (0.45–2.34)	
**SII**		0.445
<909,583.93	ref	
≥909,583.93	1.49 (0.53–4.20)	
**CAR**		0.819
<2.86	ref	
≥2.86	0.92 (0.46–1.82)	

−2 Log Likelihood = 385.79, *p* < 0.001.ref: reference.

**Table 7:** Multivariate Cox regression results of progression risk according to various clinical variables.

Variables	HR (95% CI)	*p*
**Age**		0.641
≤65	ref	
>65	1.16 (0.62–2.16)	
**Smoking**		0.831
No	ref	
Yes	0.89 (0.33–2.42)	
**Alcohol**		0.260
No	ref	
Yes	0.59 (0.23–1.47)	
**Surgery**		**0.001**
No	ref	
Yes	0.33 (0.17–0.64)	
**Stage**		**0.024**
III	ref	
IV	2.19 (1.11–4.34)	
**Chemotherapy response**		**<0.001**
Stable disease	ref	
Progressive disease	3.25 (1.31–8.08)	**0.011**
Mixed response	0.86 (0.13–5.56)	0.875
Complete response	0.48 (0.17–1.34)	0.163
Partial response	1.40 (0.40–4.79)	0.592
**Second-line CT**		**0.009**
Received	ref	
Not received	0.37 (0.18–0.78)	
**NPS**		**0.028**
Grade-0	ref	
Grade-1	3.79 (1.16–12.40)	**0.027**
Grade-2	8.05 (1.75–37.05)	**0.007**
**CONUT**		0.316
<3	ref	
≥3	0.66 (0.29–1.48)	
**SIRI**		0.526
<1890.62	ref	
≥1890.62	1.32 (0.55–3.15)	
**SII**		0.789
<909,583.93	ref	
≥909,583.93	1.14 (0.42–3.10)	
**CAR**		0.709
<2.86	ref	
≥2.86	1.12 (0.61–2.06)	

−2 Log Likelihood = 438.72, *p* < 0.001. ref: reference.

## Data Availability

The data in this study are available from the corresponding author upon reasonable request.

## Supplementary Materials

The following supporting information can be downloaded at: https://www.mdpi.com/article/10.3390/jcm14248924/s1, Table S1: Sensitivity Analysis for Prognostic Scores; Table S2: Detailed Treatment Characteristics.
