# Supplementary material for: Real-World Outcomes of Treatment Approaches and the Impact of Systemic Inflammation Markers on Survival in Patients with Locally Advanced and Metastatic Laryngeal Cancer
Source: J Clin Med. 2025 Dec 17;14(24):8924. doi: 10.3390/jcm14248924 (PMC12734149; doi:10.3390/jcm14248924)
Supplement: Supplementary file 1 [file jcm-14-08924-s001.zip › jcm-4006741-supplementary.pdf]

Figure 1A–1E. Kaplan–Meier overall survival curves according to inflammatory and nutritional indices.

(A) **NPS**: Higher NPS grades were associated with significantly reduced OS (p<0.001).
(B) **CONUT**: Patients with CONUT ≥3 demonstrated worse OS (p=0.001).
(C) **SIRI**: Elevated SIRI (≥1890.62) was associated with poorer OS (p<0.001).
(D) **SII**: Higher SII (≥909,583.93) predicted significantly shorter OS (p<0.001).
(E) **CAR**: CAR ≥2.86 was linked to worse OS (p=0.001).

Figure 1A. Overall survival according to NPS.

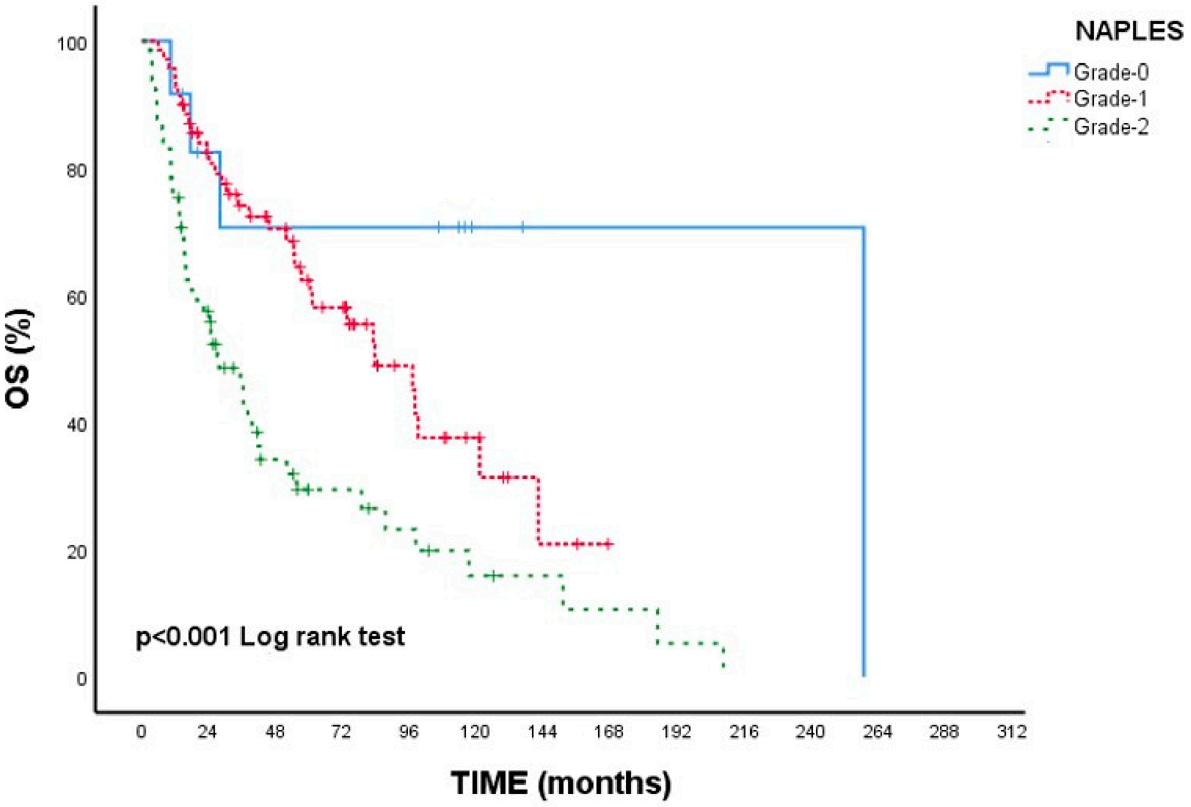

Figure 1B. Overall survival according to CONUT.

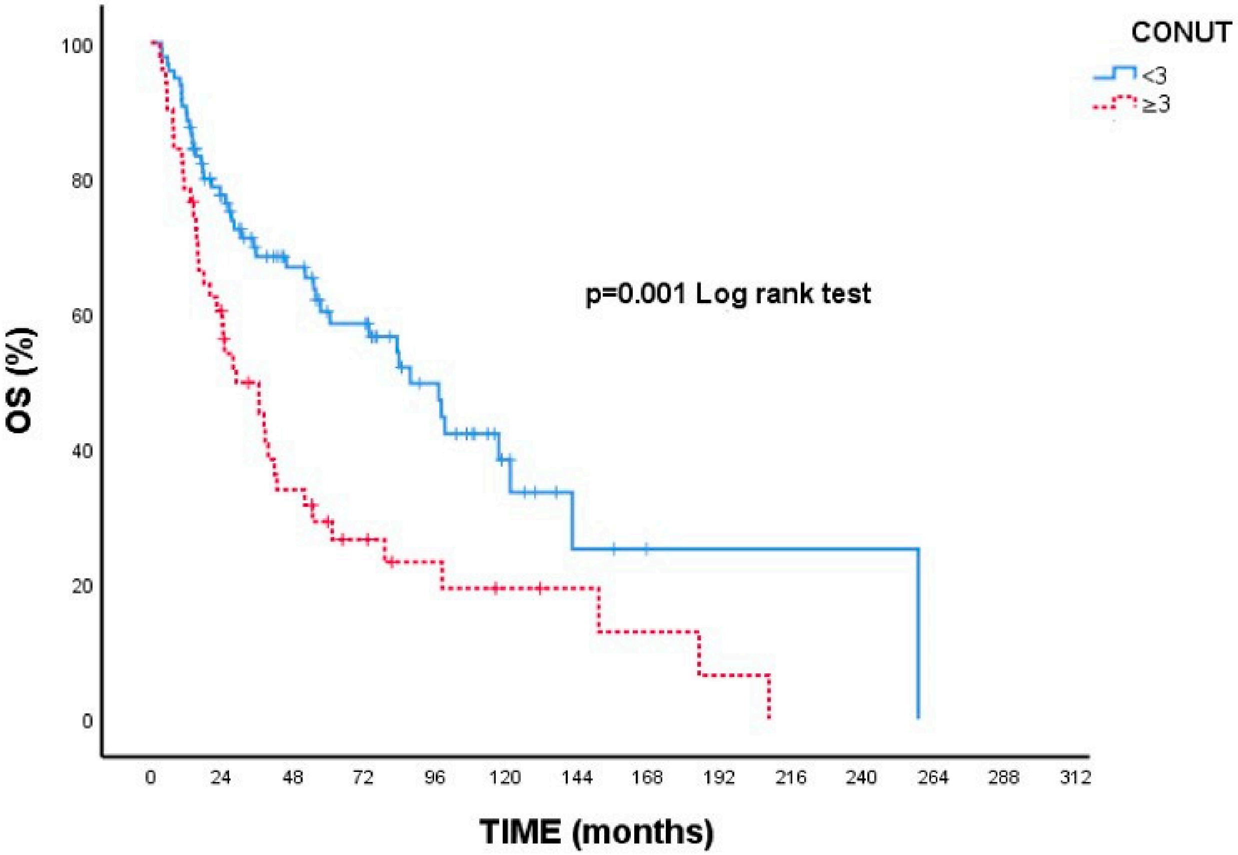

Figure 1C. Overall survival according to SIRI.

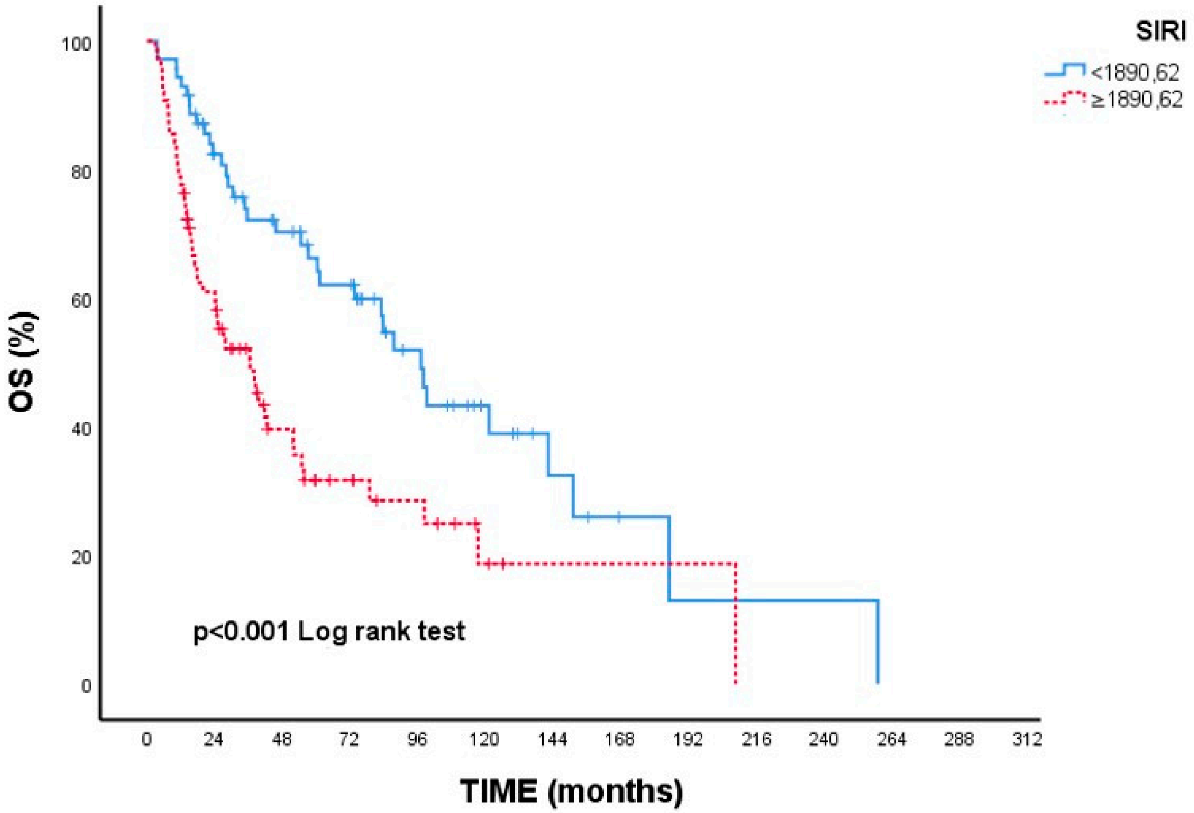

Figure 1D. Overall survival according to SII.

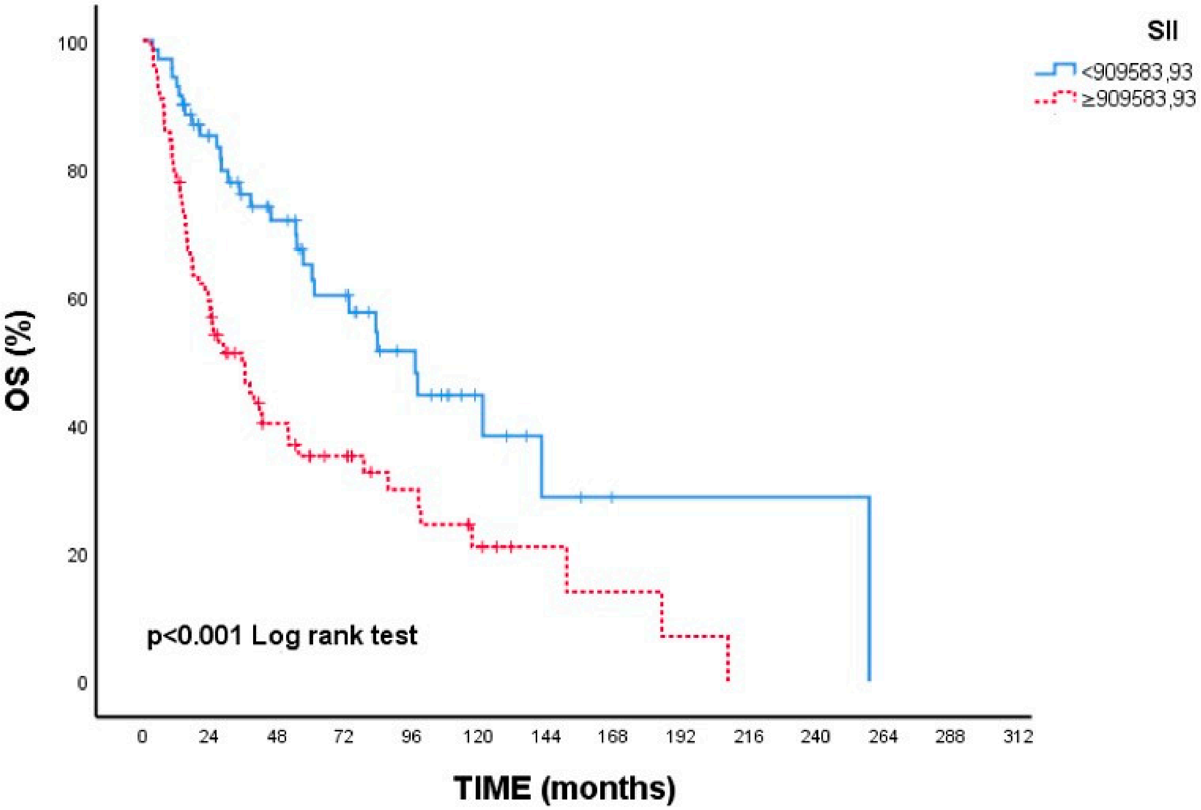

Figure 1E. Overall survival according to CAR.

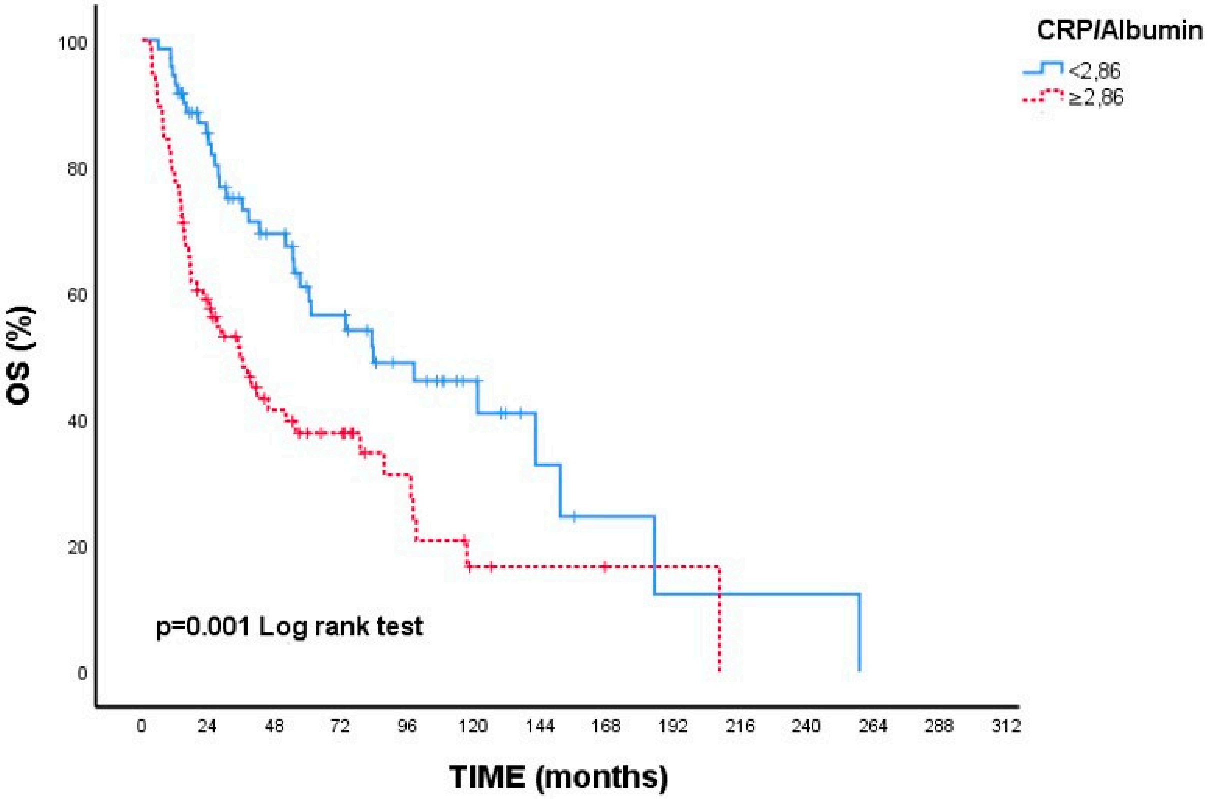

CRP/Albumin
<2,86
≥2,86
OS (%)
100
80
60
40
20
0
p=0.001 Log rank test
0
24
48
72
96
120
144
168
192
216
240
264
288
312
TIME (months)
